# Supplementary material for: The genome sequence of Astragalus glycyphyllos L., 1753 (Fabales: Fabaceae)
Source: Wellcome Open Res. 2026 Jun 10;11:347. [Version 1] doi: 10.12688/wellcomeopenres.26638.1 (PMC13324363; doi:10.12688/wellcomeopenres.26638.1)
Supplement: Peer review history

# Reviewer report: Reviewer response for version 1

**DOI:** 10.21956/wellcomeopenres.29334.r158216

**Published:** 2026-07-01

**Authors:** Daniel G Peterson

**Affiliations:** 1 Mississippi State University, Mississippi, USA

The manuscript of Leitch et al. describes the generation of sequence data for *Astragalus glycyphyllos* (Wild Liquorice). Wild Liquorice has potential medicinal value and is a representative of the largest angiosperm genus (>3000 species).  Nuclear, mitochondrial, and chloroplast genomes were assembled from sequence reads.  Additionally, mRNA sequences from *A*. *glycyphyllos* were obtained.  Of note, all sequence assembly summary metrics for the *A*. *glycyphyllos* nuclear genome surpassed the benchmark metrics for Earth BioGenome Projects.

 The authors estimated the nuclear genome of *A*. *glycyphyllos* at 1C = 850 Mb by flow cytometry which is considerably larger than their 1C = 719.74 Mb size predicted by GenoScope2.0 analysis. Further analysis of the sequence data should provide some insight into this discrepancy.

 The nuclear genome assembly was largely contained within 8 chromosome-level scaffolds.  BUSCO v5.5.0 analysis of the chromosome scaffolds suggests 97% of expected fabales genes are found within the genome.

 The authors have generated powerful data sets that can be utilized to explore the genome of Wild Liquorice and its relatives. The data (including assemblies) is high quality and is ready for biological discovery by the authors and others. The manuscript is well written and easy to understand.

Are sufficient details of methods and materials provided to allow replication by others?

Yes

Is the rationale for creating the dataset(s) clearly described?

Yes

Are the datasets clearly presented in a useable and accessible format?

Yes

Are the protocols appropriate and is the work technically sound?

Yes

Reviewer Expertise:

plant; genomics; genome sequencing; cytogenetics

I confirm that I have read this submission and believe that I have an appropriate level of expertise to confirm that it is of an acceptable scientific standard.

# Reviewer report: Reviewer response for version 1

**DOI:** 10.21956/wellcomeopenres.29334.r158831

**Published:** 2026-06-24

**Authors:** Ruslan Kalendar

**Affiliations:** 1 University of Helsinki, Helsinki, Finland

This Darwin Tree of Life genome note clearly presents a chromosome-level assembly of A. glycyphyllos (wild liquorice/liquorice milkvetch). The assembly meets the Earth BioGenome Project reference standard: combined QV 63.4; BUSCO completeness of 97.1% against fabales_odb10; contig N50 of 3.05 Mb; scaffold N50 of 84.78 Mb; and 99.71% of the sequence is assigned to the eight expected pseudomolecules (consistent with the published karyotype of 2n = 16, x = 8). The core assembly statistics are internally consistent: the eight chromosome lengths in Table 3 sum to 678.3 Mb (the stated 99.71% of the total of 680.28 Mb), and the contig/gap/scaffold relationship (398 gaps + 17 scaffolds = 415 contigs) is correct. The methods follow the well-documented, established DTOL pipeline and the relevant software versions and protocols.io references are provided. All raw data and the assembly have been openly deposited under PRJEB65706. Justifying the placement of a reference genome in Astragalus, the largest angiosperm genus, is clearly explained in the paper.

 The per-chromosome GC content in Table 3 appears to contain placeholder or over-rounded values. All chromosomes 1-6 are reported as exactly 36.50% GC, with only chromosomes 7 (38.50%) and 8 (37%) differing. It is not plausible for six independent chromosomes to share an identical value to two decimal places for real per-chromosome GC; this looks like a table-population or coarse-rounding artefact. 

 The scaffold count and span are reported inconsistently across the text and figures. Table 2 and the 'Assembly Statistics' section give 17 scaffolds and a span of 680.28 Mb, whereas the BlobToolKit snail plot (Figure 5) and blob plot (Figure 6) report 20 scaffolds and a total span of 681 Mb. The difference of three scaffolds (and 0.5 Mb) is presumably due to the inclusion of the two mitochondrial sequences and the plastid by BlobToolKit, whereas Table 2 reports these separately. Genome size and coverage figures would also benefit from reconciliation and a brief explanatory comment.

 The three independent size estimates differ considerably: flow cytometry 850 Mb (1C = 0.87 pg), GenomeScope 719 Mb and the final assembly 680.28 Mb. It is unsurprising that the assembly is 20% smaller than the cytometric estimate (given the 48% repeat content and collapsed repeats), but a sentence acknowledging and briefly interpreting the discrepancy would strengthen the report. The haploid estimate from GenomeScope is given as 719.74 Mb in the text, but the header of Figure 2 reads 'len 718,562,896 bp'.  

 The stated 'approximately 29×' HiFi coverage cannot be derived cleanly from either size estimate (21.70 Gb/719.74 Mb = 30×; 21.70 Gb/850 Mb = 26×).  

 'Two mitochondrial genomes' - please provide a biological interpretation. The note reports two mitochondrial sequences (173.12 kb and 189.37 kb). Given the well-known structural complexity of plant mitogenomes, and the fact that OATK can output multiple mitochondrial contigs, it would be helpful to state whether these represent two structural components of a multipartite mitochondrial genome, alternative isomeric configurations or two separate assembled molecules. Using wording such as 'two mitochondrial molecules/contigs' rather than 'two mitochondrial genomes' would also be clearer.

 The species epithet is misspelled in the Figure 1 caption (Astragalus glycyphillos → glycyphyllos).

Are sufficient details of methods and materials provided to allow replication by others?

Yes

Is the rationale for creating the dataset(s) clearly described?

Yes

Are the datasets clearly presented in a useable and accessible format?

Yes

Are the protocols appropriate and is the work technically sound?

Yes

Reviewer Expertise:

genomics, molecular biology

I confirm that I have read this submission and believe that I have an appropriate level of expertise to confirm that it is of an acceptable scientific standard.
